# A matter of trust: Learning lessons about causality will make qAOPs credible

**DOI:** 10.1016/j.comtox.2021.100205

**Published:** 2022-02

**Authors:** Nicoleta Spînu, Mark T.D. Cronin, Judith C. Madden, Andrew P. Worth

**Affiliations:** a School of Pharmacy and Biomolecular Sciences, Liverpool John Moores University, Byrom Street, Liverpool L3 3AF, UK; b European Commission, Joint Research Centre (JRC), Ispra, Italy

**Keywords:** Model credibility, Adverse Outcome Pathway, qAOP, Causality, Next Generation Risk Assessment

## Abstract

Toxicology in the 21st Century has seen a shift from chemical risk assessment based on traditional animal tests, identifying apical endpoints and doses that are “safe”, to the prospect of Next Generation Risk Assessment based on non-animal methods. Increasingly, large and high throughput *in vitro* datasets are being generated and exploited to develop computational models. This is accompanied by an increased use of machine learning approaches in the model building process. A potential problem, however, is that such models, while robust and predictive, may still lack credibility from the perspective of the end-user. In this commentary, we argue that the science of causal inference and reasoning, as proposed by Judea Pearl, will facilitate the development, use and acceptance of quantitative AOP models. Our hope is that by importing established concepts of causality from outside the field of toxicology, we can be “constructively disruptive” to the current toxicological paradigm, using the “Causal Revolution” to bring about a “Toxicological Revolution” more rapidly.

## Highlights

• Quantitative AOPs are toxicodynamic models based on Adverse Outcome Pathways.
• Mathematical models, e.g. Bayesian networks, can inform the quantification of AOPs.
• Model credibility is enhanced by applying the principles of causality theory.
• A causal diagram is illustrated for an AOP for Parkinsonian motor deficits.
• Computational resources to help assess causality are listed.

“*I would rather discover one causal relation than be king of Persia*” (Democritus, 430–380 BCE)

The 21st Century has seen a shift from chemical risk assessment based on traditional animal tests, identifying apical endpoints and doses that are “safe”, to the prospect of Next Generation Risk Assessment (NGRA) based on non-animal methods and led by exposure and mode of action considerations. There have been a number of drivers for this process and an undoubted catalyst is the Adverse Outcome Pathway (AOP) concept and its community of practice. A decade on from the inception of AOPs, it is easy to see that Ankley et al. [1] hit the sweet spot at the right time. Arguably, the World was in the mood for forward-looking and unifying frameworks, following the global financial crash of 2008. The vision of toxicology based on analysis of pathway perturbations, rather than finding adverse outcomes, was presented in the seminal paper “Toxicity Testing in the 21st Century” [2] at a time when new technologies were becoming increasingly available to give insights into biochemical and physiological pathways. The AOP framework presented was elegant in its simplicity, fundamental in its scientific basis and irrepressible! A decade later, the original linear, qualitative AOP concept has evolved in several ways. With more and more data being generated from emerging technologies, e.g., 2-D and 3-D *in vitro* models, batteries of *in vitro* tests, organ-on-a-chip, high-throughput and high-content screening technologies, as well as machine learning (ML) approaches being applied, the opportunities for quantifying AOPs cannot be missed.

First described by Villeneuve [3], quantitative Adverse Outcome Pathway (qAOP) models are considered a cornerstone to screen molecules, predict their points of departure from normal physiological pathways, and quantify the relationships between upstream events and downstream events, including the adverse outcome (AO). The Organisation for Economic Co-operation and Development (OECD) Guidance document on the use of AOPs in Integrated Approaches to Testing and Assessment (IATA) [4] defines a qAOP as “*an assembly of key events (KEs) supported by descriptions of how the KEs can be measured and the accuracy and precision with which the measurements are made along with key event relationships (KERs) supported by a quantitative understanding of what magnitude and/or duration of change in the upstream KE is needed to evoke some magnitude of change in the downstream KE*”. In this commentary, we describe how mathematical modelling, and in particular the use of Bayesian networks, can be used to inform the quantitative understanding of KERs. As of mid-2020, there are only a handful of models for qAOPs publicly available [5]. The practical difficulties in developing a qAOP model are many and diverse, starting with a lack of appropriate quantitative data compared to the abundance of qualitative mechanistic information. There are also challenges in integrating multiscale omics and phenotypic data to identify and map causal effects induced by toxicants. The strategy for developing a qAOP is dictated by what goes into the model and what is expected as an output from the model. At one level, quantification refers to individual KEs, which are inferred from quantitative data for the causal cascade of events. At another level, quantification of individual KERs and indeed the entire AOP can be achieved by mathematical modelling using deterministic models, or a probability distribution, or a conditional probability table (CPT) as in a Bayesian network. It is therefore reasonable to ask, what efforts are needed to advance the concept of qAOP in predictive toxicology?

Emerging approaches, or so-called New Approach Methodologies (NAMs), are increasingly considered to be the basis of NGRA, as exemplified by studies such as Baltazar et al. [6]. Recently, Knight et al. [7] have expressed a view on the actions needed to support these new approaches while Cronin et al. [8] have made a “call for action” that they be implemented. Although relevant and reliable methodological approaches are being developed and demonstrated, a key pre-requisite for their widespread application is trust. Trust in NAMs is required at various levels, from the risk manager in industry, to the regulatory scientist in a government agency, to the consumer. Trustworthiness, or credibility, is not an intrinsic property of the NAMs themselves but depends on a shared belief with all stakeholders [9]. As we move towards an Artificial Intelligence (AI)-driven society, understanding how we can build trust will give stakeholders confidence and reduce the conspiracy theory laden “fake news”. Finding effective and efficient ways of building trust in NAMs is crucial, irrespective of whether we aim for incremental change, or if we consider that an element of “disruptive thinking” is required to increase the acceptability of new methods [10]. We need to take risks and shake up the *status quo* to make progress, but changes made in the name of progress must be broadly accepted and sustainable. The question is how long will it take for innovations to diffuse, given the conservative tradition of the science?

An essential requirement for trustworthy models is to demonstrate causality. Reproducible and robust results cannot be achieved with observations or random patterns alone [11]. It is about formulating hypotheses about the underlying mechanistic relationships and testing these hypotheses through carefully designed experiments, typically, in an iterative manner. Thus, models that are fit-for-purpose instead of solely providing predictions are more likely to be credible (acted upon). For instance, Musuamba et al. [12] advocated for well described and clearly stated scientific questions as part of a risk-informed evaluation framework for the credibility assessment of *in silico* models for drug development. In terms of the toxicology supporting chemical risk assessment, causality is conventionally represented by the direct association with the mechanism of action producing the AO. This explains the popularity of the (q)AOP paradigm, which is firmly based on mechanistic thinking. Whilst this understanding of causality is obvious to many in the area of toxicological risk assessment, it is worthwhile to consider the subject of causality in more detail, especially as it forms an integral part of many mathematical approaches to qAOP development. Here, we highlight in particular the utility of Bayesian theory, which has the potential to become one of the dominant modelling approaches.

Bayesian statistics is an approach to data analysis and parameter estimation based on Bayes’ theorem [13]. It summarises the results as probability distributions on the parameters of the model based on observed quantities [14]. The core element in the Bayesian framework is the prior that reflects the knowledge/belief about the parameters before the data collection step. The usefulness of the prior is that it can be updated once new information becomes available [13]. For instance, quantification of an AOP with little information can be started with some prior judgement based on domain expertise, which can be updated to reflect new evidence, for example new data generated by NAMs. It is not sufficient to collect any data, but rather generate and interpret relevant data to unveil cause-effect relationships for evidence-based decision making. The combination of causal principles with Bayesian inference allows for the estimation of the effect size of model parameters, e.g., as a result of an intervention, including evaluation of all possible combinations of perturbations that in practice is rarely achievable.

To better understand causality, we should not restrict our thinking to our pre-conceptions of toxicology for risk assessment. The science of causality has been brought to light by Judea Pearl who, in his recent book “*The Book of Why*” written with Dana Mackenzie [15], tells the “silent history of cause and effect”. Pearl and Mackenzie explain how the “Causal Revolution” is the new paradigm that is much needed to progress computational modelling, especially in the context of artificial intelligence (AI). The importance of understanding causal relationships is evidenced by the realisation that traditional statistical thinking fails to address real-world causal processes. Since causality is the direct relationship of cause and effect, this can only be established by evidence, but often this evidence requires painstaking efforts to obtain. No wonder causal reasoning is the neglected child of computational modelling and AI, technologies that are possibly promoted more by hope and hype than reality. At a time when there are increased opportunities for computational modelling in toxicology and the sustainable management of chemicals [16], it is surely time to step back and consider what causality means in the “new age” and why it will be crucial in a society requiring evidence, and not just a convincing narrative about what events trigger other events.

In this commentary, we explore the key role of causality in developing models, as proposed by Judea Pearl, to facilitate the development, use and acceptance of qAOP models. Our hope is that by importing established concepts of causality from outside the field of toxicology, there will ultimately be a wider acceptance of models by all stakeholders. The intention is to provide a stimulus to be “constructively disruptive” to the current toxicological paradigm, i.e., how we can use the “Causal Revolution” to bring about a “Toxicological Revolution” more rapidly.

**Fig. 1:**
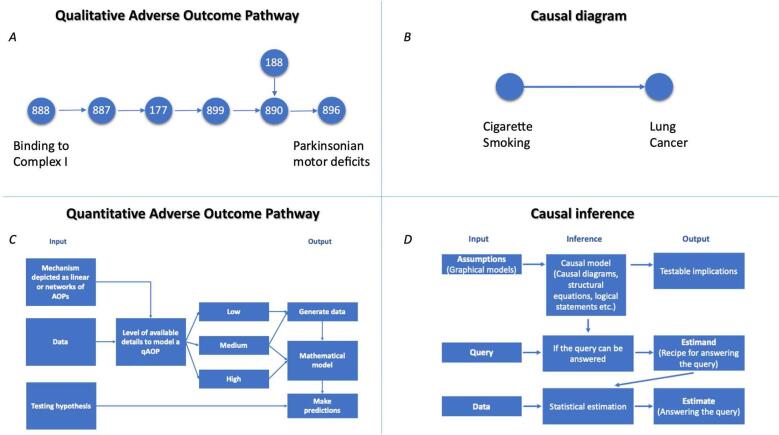
A. The AOP for Parkinsonian motor deficits is taken as an example to underline one of the characteristics of a qAOP model, mainly understanding the cause and effect in the context of predictive toxicology (https://aopwiki.org/aops/3). Numbers represent the indices of the events in the OECD AOP-Wiki Knowledge Base available at https://aopwiki.org/events/XXX, where xxx is the index in the node. B. A causal diagram representing the linkage between cigarette smoking and lung cancer. C. Scheme for the general process of qAOP model development and application. Depending on the available level of resources, an AOP can be used to generate data or model quantitatively to make predictions and test a hypothesis. D. The causal inference engine was proposed by Judea Pearl as described in the text and is taken from [15].

While a qAOP model can in principle be data-driven [5], we propose that it should be a fundamental requirement for a qAOP model to express the causal relationships between a stressor and the toxicity endpoint/AO of interest. The stressor is typically a chemical but could also be a pathogen such as a virus [17]. As an example of a chemically induced AO, Bal-Price et al. [18] compiled evidence showing that a brain concentration of 20–30 nM of rotenone in rats leads to approximately 53% of inhibition of complex l of the mitochondrial respiratory chain. This, in turn, leads to an approximately 20–53% decrease in respiration rate and, possibly more significantly, an approximately 20–60% decrease in ubiquitin proteasomal system activity which is involved in neuronal loss and motor impairment, the latter being responsible for Parkinsonian motor deficits (Fig. 1A). These statements were derived from experimental studies combined with domain knowledge, conceived within a qualitative AOP framework. Tools to translate such assumptions into mathematical models to simulate the magnitude by which a downstream key event is altered, or perturbed, by a change in an upstream key event will advance the qAOP framework and make it applicable. In this context, the prior in the Bayesian model can serve as a bridge between what is known and what else is required to validate the mechanistic assumptions.

To understand the fundamental principle of causality in an AOP, let us consider whether the causality is known or assumed, and whether the AOP framework itself allows us to think in a sufficiently causal way. Although there is no “big” theory of toxicology [19], an AOP can be regarded as an established “small” theory. Pearl refers to the “causal diagram” as the way to represent our scientific knowledge about a variable of interest, also termed a directed acyclic graph (DAG). Thus, causal diagrams consist of variables (nodes) or quantities and arrows (edges) that indicate known or suspected causal relationships between those variables (Fig. 1B). Pearl advocates the use of such diagrams because: a) they are easy to draw and comprehend; b) they can estimate all sorts of causal relationships – simple or complicated, deterministic or probabilistic, linear or nonlinear; and c) most importantly, they allow for the storage of information for future reference and application. Additionally, DAGs are models representing how we think the world, or process of interest, works. Once written, a DAG helps to identify all testable implications and establish whether the model derived is compatible with the data. Furthermore, a causal diagram is a Bayesian network, a term coined by Judea Pearl in 1985. Each arrow implies a direct causal relation, or at least the possibility of one, in the direction of that arrow. Pearl confesses that “*[he] wanted Bayesian networks to operate like the neurons of a human brain; you touch one neuron, and the entire network responds by propagating the information to every other neuron in the system*” [15]. Furthermore, Bayesian networks are efficient in coping with contradictory and uncertain data that can be implemented on modern computer platforms. Other advantages of Bayesian networks are that: a) they are transparent and understandable compared to other computational techniques, e.g., deep neural networks; b) they allow every step to be followed, pinpointing how and why each piece of evidence changed the network’s beliefs; c) there is no need to intervene to tell the network how to evaluate a new piece of data; d) updating can be done very quickly; and e) the network is integrative, which means that it reacts as a whole to new pieces of information.

However, Bayesian networks should be developed and interpreted with care. Depending on how they are constructed, they are not necessarily causal. There is also a need to avoid any ambiguity in the way the node is classified into states such as high, medium, or low. In the case of larger networks, where a node has multiple “parent” nodes, there is also the practical challenge of specifying the conditional probability tables (CPTs). As Pearl points out, “*while probabilities encode our beliefs about a static world, causality tells us whether and how probabilities change when the world changes, be it by the intervention or by act of imagination*” [15]. Pearl adds that “*the main differences between Bayesian networks and causal diagrams lie in how they are constructed and the uses to which they are put. A Bayesian network is literally nothing more than a compact representation of a huge probability table*” [15]. In other words, a Bayesian network can tell how likely one event is, given an observed one, while causal diagrams can answer interventional and counterfactual questions. Interventional questions ask what effect an intervention will produce on the observed variable. For example, does chemical X alone, or in combination with a chemical Y, induce adverse effects? Counterfactual questions ask “what if” something else had happened, referring to a process analysed retrospectively. In the counterfactual case, Pearl explains that “*we imagine a different scenario in order to change the circumstances being analysed*” [15]. For example, what if a person were exposed to chemical X, but not under the specified conditions, would the same adverse effect be observed? In other words, counterfactual questions allow us to assess situations that cannot be observed or measured in real-life, or in the past, due to ethical considerations or incapacity to perform such experiments.

Pearl also comments that “*with Bayesian networks, we had taught machines to think in shades of grey, and this was an important step toward humanlike thinking. But we still couldn’t teach machines to understand causes and effects*” [15]. Hence, the science focused on identifying patterns in data rather than understanding the reason for those patterns. Given this limitation, it becomes obvious why an understanding of Pearl’s concepts is relevant to the AOP framework. They provide the conceptual basis for developing and quantifying an AOP, especially through Bayesian networks or similar approaches. Additionally, the causality methodology proposed by Pearl helps to answer both types of “why” questions: the straightforward one, when we seek to know the cause of toxicity (i.e., the stressor and the potential adverse effect), and the more challenging one when we want to understand the mechanism itself.

The development of a qAOP model is hypothesis driven. AOPs give us the opportunity to test a hypothesis to examine the causal evidence for an adverse effect with human or ecological relevance. We consider that mechanistic modelling, which implies the use of empirical dose-responses, Bayesian networks and systems biology, to be most appropriate for such purposes. For example, a simplified AOP mechanistic model linking thiol oxidation to chronic kidney disease through oxidative stress and mitochondrial disruption was quantified by Zgheib et al. [20], allowing three quantitative approaches to be compared [20]. Additionally, this qAOP model allowed for the evaluation of different levels of exposure of a chemical tested over time and the derivation of chemical-independent key event relationships by inversion of the empirical model applied. Another example of accounting for causality represents the quantification of a simplified AOP network for developmental neurotoxicity [21]. The AOP network was constructed from a set of linear AOPs, which were available in the OECD AOP Wiki Knowledge Base and developed following the principles of the weight of evidence analysis. The hypothesis included key events identified as most connected based on a topology analysis of the network. The model was constructed using NAM-generated data, and a Bayesian parametric approach was employed to evaluate compounds for their probability of following the causal relationships and inducing the biological events. In other words, a qAOP model serves as a tool to translate descriptive qualitative assumptions into the quantitative predictions of an AO in hazard and risk assessment, as shown in Fig. 1C.

Considering in more detail how to develop a qAOP model, we need to think about the (key event) data underlying the relationships. Pearl asserts that “*data are profoundly dumb*” because they cannot tell us the “*why*” [15]. Therefore, as Pearl indicates, “*causal questions cannot be answered from data alone, it needs to formulate models that generate the data to understand their patterns*” [15]. Also, Pearl emphasises that “*data interpretation means hypothesising on how things operate in the real world*” [15], and even though conclusions can be drawn with only partial information, and not necessarily knowing every causal relation between the variables of interest, a minimum causal hypothesis is always required. Importantly, Pearl makes a distinction between two types of data interpretation: deduction – reasoning from hypothesis to conclusion, and induction – reasoning from evidence to a hypothesis. This is akin to what is sometimes referred to as the forward problem versus the inverse problem [22]. Furthermore, Pearl’s methodology is based on Bayes’ rule and consists of several steps: (1) formulate a hypothesis, (2) deduce a testable consequence of the hypothesis, (3) perform an experiment and collect evidence, and (4) update your belief in the hypothesis. Consistent with this methodology, the development of a qAOP does not require pattern recognition but rather relies on the measurement of relevant data by using assays associated with key events.

If we accept the need to consider causality in predictive toxicology models, and in particular qAOPs, then we need means to evaluate that causality. As we move to the holy grail of regulatory acceptance of models and their predictions, the demonstration of causality within the model becomes paramount. Traditional approaches for evaluating causality in toxicology follow the criteria formulated by Austin Bradford Hill in 1965 who attempted to summarise the arguments for the causal linkage of cigarette smoking to lung cancer (Fig. 1B) [23]. This cause-effect relationship led to several achievements including: (i) the establishment of randomised control trials methodology conducted by Doll and Hill to compare a treatment group (patients with diagnosed cancer) to a control group (healthy volunteers) [24]; (ii) Cornfield’s inequality that described the hypothesis of the presence of a smoking gene that makes the difference of developing lung cancer and which has driven the methodology of sensitivity analysis, and most importantly; (iii) Hill criteria that helped to summarise the evidence and which are now widely utilised. More recently, the so-called “modified Bradford Hill” criteria have been used as the basis of a framework for evaluating KERs for the development of qualitative, semi-quantitative and quantitative weight-of-evidence qAOP models [25], [26], [27]. This is because regulatory toxicologists cannot rely solely on the NAM-generated data without a mechanistic understanding of causal linkages established between exposure to chemicals, or other types of stressors, and the health effect of regulatory concern. However, an important question is whether the Hill criteria, in their original or modified form, are adequate to demonstrate causality. Pearl describes the Hill criteria as being “*qualitative patterns of statistical trends*” and asserts that Hill himself called them “*viewpoints*” and not requirements. Pearl emphasises that the “*Hill’s “viewpoints” are still useful as a description of how a discipline comes to accept a causal hypothesis, using a variety of evidence, but they came with no methodology to implement them. Each scientist just has to decide for him- or herself. But gut decisions can be wrong, especially if there are political pressures or monetary considerations*” [15]. For example, the consistency or strength of the association that comes with the Hill criteria by itself may prove nothing “*if thirty studies each ignore the same confounder, all can easily be biased*” [15]. Put simply, a confounder represents a common cause of two potentially independent variables. In the context of an AOP, we can think of confounders as being the modulating factors such as a person’s age, diet, genetic predispositions etc. The concept of confounder can help understand the difference between causal reasoning and causal inference, the former being what we want to assess, and the latter what we actually assess using statistical methods. While causal inference cannot be carried out in the absence of confounders, causal reasoning can adjust for the effects of confounding variables.

The OECD programme on the development of AOPs ensures that the modified Bradford Hill criteria (i.e., essentiality, biological plausibility, empirical evidence assessment) are followed. However, few qualitative AOPs have been endorsed so far, due to the amount of time and work required to gather the support to substantiate the building blocks, i.e., KEs and KERs. Pragmatic approaches are needed for the development of robust AOPs, as discussed by Svingen et al. [28]. Methods for causal discovery and reasoning have the potential to facilitate the knowledge assembly, thereby supplementing the weight of evidence analysis broadly accepted and practised by the scientific community and regulatory toxicologists. For example, Lazic et al. [29] applied Bayesian mediation analysis to a problem in toxicology related to animal testing of novel molecules, namely the organ weight changes due to chemical damage that might be influenced by the changes in the overall body weight. A challenge greater than the practical details of data collection or statistical analysis remains for the stakeholders to agree on the underlying (q)AOP relationships.

Importantly, Pearl posits three levels of causation, which he calls the “Ladder of Causation”, referring to the human cognitive ability: seeing, doing, and imagining. It includes: (1) associations or purely statistical relationships, (2) interventions, (3) counterfactual or explanatory questions. Climbing to the top of these three rungs, Pearl speculates that we could make machines think: “*a causal reasoning module will give the machines the ability to reflect on their mistakes, to pinpoint weaknesses in their software, to function as moral entities, and to converse naturally with humans about their own choices and intentions*” [15]. This describes a future world in which AI is genuinely “intelligent” rather than being a buzz word for today’s machine learning techniques. As computational toxicology ascends Pearl’s Ladder, we can imagine AI supporting chemical risk assessment in a multiplicity of ways, thereby addressing the complexity of the exposure to chemicals and the associated risks to human health [30].

Thus, we evaluate causality not only in qAOPs but also throughout the full toxicological paradigm (toxicokinetics and toxicodynamics), accepting the Hill criteria as a starting point, or framework, on which to hang our evidence. Understanding the limitations of the Hill criteria takes us a step closer to a more ideal approach; embracing a deeper treatment of causality offers insights into where to go next. Moreover, a range of algorithms already exists to facilitate the integration of causal reasoning into toxicology, as summarised in Table 1.

**Table 1:** List of available algorithms to help to assess causality.

Package Name	Programming Language	URL
CausalLift	Python	https://github.com/Minyus/causallift/
CausalML	Python	https://github.com/uber/causalml
DoWhy	Python	https://github.com/Microsoft/dowhy
EconML	Python	https://github.com/Microsoft/EconML
pylift	Python	https://github.com/wayfair/pylift
pymatch	Python	https://github.com/benmiroglio/pymatch
causaleffect	R	https://cran.r-project.org/web/packages/causaleffect/
causalGAM	R	https://cran.r-project.org/web/packages/CausalGAM/
dagitty	R	https://cran.r-project.org/web/packages/dagitty/
ggdag	R	https://cran.r-project.org/web/packages/ggdag/
mediation	R	https://cran.r-project.org/web/packages/mediation/
pcalg	R	https://cran.r-project.org/web/packages/pcalg/
uplift	R	https://cran.r-project.org/web/packages/uplift/

Hence, the “causal inference engine” proposed by Pearl to handle causal reasoning aligns perfectly with the stages in the development of a qAOP model. Pearl presents his blueprint as a diagram similar to a decision tree in which inputs enter the inference engine and produce the outputs (Fig. 1D). It has three different kinds of inputs: assumptions given by the knowledge, queries and data. The first output is a yes/no decision to a query; if the answer is yes, an “estimand” is produced. The second output is a mathematical formula for generating the answer from hypothetical data. The third output is produced after the data are entered, which is the answer or “estimate”, including statistical estimates of the uncertainty. Pearl adds “*this uncertainty reflects the limited size of the data set as well as possible measurement errors or missing data*” [15]. Rather than relying simply on the subjective application of the Hill criteria, is it time to embrace causality theory more extensively to quantify the uncertainties in qAOPs?

So how far have we come and what lies in store? Modelling, in all its glory and infamy, is fundamental to the paradigm change dictated by 21st Century Toxicology. At present, qAOPs are in their infancy, although like any infant, much is expected as they flourish and mature. We believe, in its essence, a qAOP model is based on two main considerations: namely an understanding of causal relationships, and the testing of hypotheses. Hence, a qAOP model can be considered a causal model to predict the results of an action (e.g., for an environmental chemical) or intervention (e.g., for a drug). However, big data tempt us look for correlations and associations instead of causality. Additionally, most of the ML techniques applied, even though exploiting independent and dependent variables, test/training and validation sets, are trained to learn from experience. But they do not infer causality of the included variables. In the context of a qAOP model, causality is currently informed by the qualitative linear or network of AOPs that promote a structured account of a stressor-induced mechanism of action assessed using the modified Hill criteria. Importantly, a qAOP model requires a considerable amount of empirical data to predict the adverse effects quantitatively. Unfortunately, the available data are typically inadequate for modelling purposes. However, causal diagrams can guide the design of appropriate experiments to generate new data and verify the assumptions of the model. Causal models can, and must, serve as a basis for the development of a robust, transparent, comprehensible and reproducible AI. More efforts are needed to shift towards understanding the causality rather than deriving data-driven models – for toxicology this means building models on mechanisms of action. Hence, we need models based on causal assumptions to draw sound and actionable conclusions. Going beyond predictive toxicology, causal models can help formulate and prioritise assessment questions in a transparent manner. This could be in the context of a retrospective analysis of the costs and benefits of a historical policy intervention (e.g., banning a certain chemical) or anticipating the potential consequences of a new policy intervention (e.g., introducing a new chemical onto the market). In both scenarios, the ability to answer counterfactual questions is a key part of causality reasoning.

One thing is certain, the causal revolution initiated by Judea Pearl is spreading. It is not only emerging in epidemiology, sociology, and economics but will also find its way into predictive toxicology, where it can contribute to the development of qAOP models. A qAOP model has the power to computationally model the causal linkages, i.e., KERs, in a transparent manner, making use of available data and applying a range of methodologies. Moreover, the model makes the data accessible, interpretable and (re)usable, hence, it has the capability to transform decision-making on chemicals. Hopefully, qAOPs will one day be accepted as more than a screening tool. The combined use of physiologically based kinetic (PBK), quantitative structure–activity relationship (QSAR) and qAOP models could, in principle, be used for a definitive chemical risk assessment.

## Declaration of Competing Interest

The authors declare that they have no known competing financial interests or personal relationships that could have appeared to influence the work reported in this paper.
